# Network analysis can provide useful insights for building resilience in social-ecological systems

**DOI:** 10.1007/s13280-025-02338-y

**Published:** 2026-02-27

**Authors:** Paul Doehring, Vanessa M. Adams, Natalie Stoeckl

**Affiliations:** 1 Tasmanian School of Business and Economics, University of Tasmania, Hobart, Australia; 2 School of Geography, Planning, and Spatial Sciences, University of Tasmania, Hobart, Australia; 3 Centre for Marine Socioecology, Hobart, TAS Australia

**Keywords:** Adaptation, Community adaptation, Participatory mapping, Resilience, Social-ecological action situations, Social network analysis

## Abstract

Communities across the world need resilience to adapt to changing circumstances. In this paper, we blend focus group work and network analysis to assess adaptive capacities and potential resilience, in a peri-urban community in Hobart, Tasmania, Australia. We used focus groups to gather information about core social and environmental features of the community and then constructed group mental models (a diagrammatic representation of how the community works). We asked participants to identify drivers (presses and pulses) of change, and to use the mental models to show how externally imposed changes might impact core features. To complement this qualitative data, network analysis was used to quantitatively identify core features, based on centrality, and to test for redundancies—assessing the likelihood of the system remaining resilient if a core feature were removed. The empirical results highlight the significance of networks’ contributions to local resilience and provide new pathways for conceptualizing change in social-ecological systems. Future research based on this case study could be scaled up and applied in many situations to improve our understanding of ways to maintain resilience at multiple geographic scales.

**Supplementary Information:**

The online version contains supplementary material available at 10.1007/s13280-025-02338-y.

## Introduction

With global environmental and social change becoming increasingly common, communities and decision makers alike must respond to changes such as climate change, pandemics, biodiversity loss, economic shocks, and geopolitical transformations (Biggs et al. 2011; Steffen et al. 2015; Folke et al. 2021; Rockstrom et al. 2023). Such changes may be slow moving (presses) or rapid (pulse) and originate from both the social and ecological realms (Collins et al. 2010). Examples of presses include climate change and changes in population demographics, while pulses represent relatively fast-moving short-term discrete events such as bushfires and political elections.

The interrelationships among slow and fast changes creates press–pulse dynamics (PPD) that illustrates the effect that human behavior has on social and ecosystem services and processes (Collin et al. 2024), thus resulting in social-ecological interactions (Levin et al. 2012; Folke et al. 2016; Preiser et al. 2018; Biggs et al. 2022). The way in which the environment ‘responds’ to a change (through ecological adaptations) will have impacts in the social/human system (e.g., climate change will almost certainly cause rising sea levels damaging livelihoods, infrastructure, and nations). So too will human responses (social adaptations) have repercussions elsewhere (Liu et al. 2013; Harangody et al. 2022). Critically, such ecological and social responses or adaptations do not always improve the situation: They can either mitigate or further exacerbate initial impacts possibly pushing the system into an undesired state which may be even more susceptible to disturbances (Silva et al. 2010; Tenza et al. 2017). Adaptations may thus either help maintain or return the system to a similar state (Folke et al. 2004; Anderies et al. 2013; Folke 2016), or instead push the system (deliberate or not) from one state to another (Walker et al. 2004; Walker 2020; Colloff et al. 2021; Scordato and Gulbrandsen 2024).

It is therefore important to understand how social-ecological systems adapt, persist, and evolve in response to changes (either rapid or slow). For our purposes, we assume that a system is resilient if it is able to navigate change by either absorbing a disturbance or avoiding an undesired change; in other words, we consider here resilience to be the property of ‘changing in order to not be changed’ (Adger 2000; Fath et al. 2015; Walker et al. 2020). In the context of press–pulse dynamics, resilient systems are likely to emerge over diverse spatiotemporal scales, with adaptation planning occurring at nested scales from global to local (Adams et al. 2017). However, adaptation responses are necessarily local and a function of unique conditions—social, cultural, political, and environmental—of place. A community’s capacity for overcoming adverse impacts of change, such as bushfires and floods (Maguire and Hagan 2007; Harangody et al. 2022) or political regime change (Orach and Schlüter 2016), is shaped by social capital, redundancy, and adaptive capacity. These three features interact with each other to help build resilient communities.

*Social capital* describes relationships of trust, reciprocity, and exchange among individuals in a community (Adger 2003). The strength of social capital may foretell how well societies solve collective action problems such as responding to crises and disruptions (Aldrich and Meyer 2015). *Redundancy* in a social-ecological system is where there are buffers or backups within the system; there is a ‘diversity of pathways’ to maintain system function, e.g., trade networks (Kharrazi et al. 2020). *Adaptive capacity* focuses on the ability of actors in a given system to respond and adjust to change (Walker et al. 2004; Folke et al. 2010; Park and Bieling 2021). Adaptive capacity requires the aid of social capital. For example, see Wardropper et al. (2025) where local knowledge and volunteers play a vital role in adapting to wildfire in Greece or Doncieux et al. (2025) who show the importance of local knowledge when building crop diversity in the Gaillac wine region in France. All three of these features help create a cohesive understanding of SES as a dynamic complex system, particularly as they change over space and time.

### Theoretical framework and general analytical approach

Building upon the Ostrom’s original social-ecological system (SES) framework (Ostrom 2007, 2009; McGinnis and Ostrom 2014), Schlüter et al. (2019) put forward the complementary idea of a social-ecological action situations framework (SE-AS). The SE-AS framework provides a means for investigating how social and ecological systems come together, interact, and respond to changes (Schlüter et al. 2019). The SE-AS framework differs from Ostrom’s SES framework in that it emphasizes social-ecological interactions (as opposed to solely social interactions) and the outcomes that ‘emerge,’ across time and space, from those interactions (Schlüter et al. 2019; Herzog et al. 2022). SE-ASs thus explicitly account for the possibility of deep connections between humans and their environment (and nature) and the contribution this makes to individual and community well-being (Borish et al. 2022; Harangody et al. 2022; Knaps et al. 2022). There are many available frameworks for understanding complex SESs that are equally relevant to and applied to—SE-AS (Walker et al. 2004; Gain et al. 2020). These include network analysis (Bodin et al. 2019), game theory (Ostrom 2009; Balbi et al. 2020), agent-based modeling (An 2012; Filatova et al. 2013; Lippe et al. 2019), system dynamics (Hossain et al. 2020), integrated assessments (Lázár et al. 2020), and coupled model frameworks (An 2012; Filatova et al. 2013). Of particular interest in this study are methods that assess networks—since SES are, by definition, networks.

The idea of using networks to study resilience in SES was discussed early in the twenty-first century (e.g., Janssen et al. (2006) and Walker et al.(2002)) and was further developed empirically through use of social networks analysis (SNA) (Salpeteur et al. 2017). SNA provides a useful tool to help analyze and synthesize specific observations and conditions of a locale’s SES and the interactions behind it. SNA has shed light on aspects of resilience and adaptive capacity such as recovery after natural disasters (e,g. Hurricane Katrina in the USA (Doerfel et al. 2013), the Kaikōura earthquake in New Zeeland (Cradock-Henry et al. 2019)), resilience in agricultural production systems (Gaillac wine region in France, (Doncieux et al. 2025)), and management and governance of protected areas (Saint Llorenc del Munt, Spain (Calvet-Mir et al. 2015)). Other avenues of exploration with SNA involve studying spatiotemporal dynamics and their respective responses. Luo et al. (2024) use social network analysis to measure the effects of village leadership on social networks in rice fields of southwest China exploring four collective action problems (forest–village–terrace–water) that village leaders use govern and influence the subsystem of the ‘Hani Rice Terrace SES’. Their research shed light on microscale interactions and how adaptations to transitions and governing challenges can have broader implications of cultural or ecological resilience (Luo et al. 2024).

However, to our knowledge, no existing methodological approach or framework has been designed to explicitly consider both the coupling of the social and ecological domains and the way systems adapt over time in response to both presses and pulses originating from either the social or the ecological subsystems. This paper thus builds upon this previous work, providing a proof-of-concept test of a novel method for considering resilience in the face of both presses and pulses. Its novelty involves, first using focus groups to first gather information about core social and environmental features of the neighborhood, asking participants to create SES maps to show how those features are connected. It then qualitatively analyzes discussions about adaptations to change in the face of both presses and pulses, and uses network metrics to test for likely redundancies within constructed the SES.

**Fig. 1:**
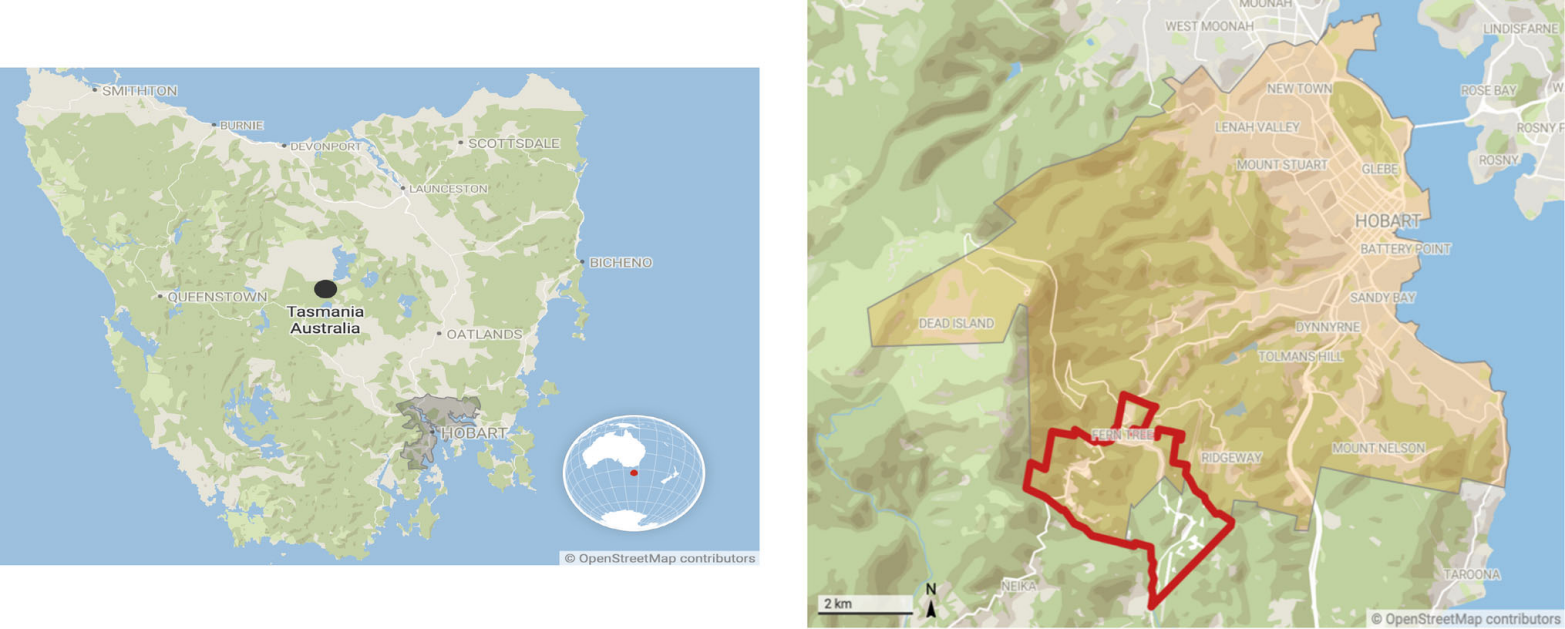
**A** Map showing case study area (Hobart), Tasmania, Australia, **B** map of greater Hobart area, with Fern Tree highlighted

Recognizing that it is too significant a task to fully investigate changes and adaptations in both the social and ecological realms at multiple geographic scales, over long time periods, this paper describes a pilot study that considers social adaptations to change at local scale. Our overarching aim is to test this methodological proof of concept to determine whether the methods are able to generate information about what makes local communities ‘resilient’. To this end, we focus our study on a local community (Fern Tree) outside Hobart, Tasmania, Australia (Fig. 1), and our investigation specifically looked for evidence of ‘resilience’ in a local SES. We meet our aim by analyzing data that is collected through focus group discussions, to answer the following research questions:

i. What are key features of the local (Fern Tree) SES? How do locals map these features into a network?
ii. How are these features related?
iii. What are some of the core drivers of change (in key features)?
iv. How might the community adapt to future change?
v. What might happen if a core feature were to ‘disappear’?

Our discussion synthesizes insights from the individual investigations, to consider the extent to which our methods collectively generate insights about social capital, redundancies and adaptive capacities—all critical parts of resilient systems. We also discuss ways in which insights from this pilot could be scaled and/or transferred to other contexts. This pilot thus demonstrates a novel way of blending focus group work and network analysis to learn more about how resilience building occurs at local scale. This information could prove useful for others, when seeking to learn how to encourage positive adaptations that build resilience in other communities, and at other scales, thus ultimately contributing to global adaptation, resilience, and sustainability goals (e.g., the SDGs) (Scown et al. 2023).

## Materials and methods

### Study area

The empirical part of this study was carried out in a small community, Fern Tree, a peri-urban area outside Hobart, Tasmania. The neighborhood consists of around 763 residents in the foothills of Kunanyi/Mount Wellington and is located approximately 10 km from the Hobart city center (Fig. 1). The demographic makeup includes a mix of 47% male and 53% female with a median age of 45. Across the landscape, there are 305 private dwellings with 227 families living in them with an average of 2 children per household. The annual household income is around 100 K AUD (67 K USD) (Australian Bureau of Statistics 2022).

Fern Tree is exposed to both ecological and social ‘shocks,’ making it an ideal location for studying complex system dynamics. It sits within a state park, an area prone to be hit (and significantly impacted) by bushfire—the last major bushfire to sweep through and devastate the area having been in 1967 (Black Tuesday). There are also social pressures—a potential significant change that could directly affect the residents of Fern Tree, being the possibility of cable car being built on Mount Wellington, which could impact local lifestyles and the surrounding environment (Australian Associated Press 2022, MWCC 2023). Here, the main concern for locals would be traffic and the pressures this would have on community access as well as nature impacts. Although previous studies have examined the resilience of Tasmanians (including Fern Tree residents), most of these have focused on just one aspect of a problem such as preparedness for bushfires, community education/well-being, or coastal governance of the local SES due to climate change (Auckland and Kilpatrick 2021; Lucas et al. 2022; Clement et al. 2024). This pilot study aims to build a framework and more cohesive understanding of social resilience building around the many factors that are in play in a SES in terms of both the natural (environmental) and social (community) features.

### Data collection and analysis

We used focus group elicitation to collect data (ethics approval H27215). The recruitment of participants for the focus groups involved targeted advertisements to reach community members in the community newsletter, which is disseminated monthly, posting on the community social media site as well as posting flyers through highly trafficked areas, e.g., local tavern, trail heads, and bus stops. The discussions were held at the Fern Tree Community Centre and individuals were paid with a $45 dollar gift card for their time. We had sufficient interest to run two focus groups: the first (FG1) comprised 2 men and 3 females; and the second (FG2) comprised 1 male and 1 female. While this sample is not fully representative of the whole community, with participants in their mature working age and/or retired stage of life (thus slightly older and more educated than the average household in Fern Tree; ABS 2021), we do think this can give us some insight on the legacy or functions that Fern Tree has to offer. Due to the passive recruitment methods used, we do not know what proportion of the 763 residents of Fern Tree would have seen and thus ‘received’ our invitation to participate and cannot estimate response rates. We recognize the limitation of our small sample size and thus emphasize that this endeavor is best considered a ‘pilot’—a prototype and trial of methods used to demonstrate how focus group information can be integrated with network analysis to learn more about neighborhood resilience.

Questions and activities within each focus group were designed to collect data relevant to each of our core research questions, as detailed below.

#### What are key the features of the local SES?

We first sought to identify the ‘most important’ features of the social-ecological system. We did this by asking participants to talk about what they liked (or disliked) most about living in Fern Tree. To begin, the facilitator asked participants, ‘What is important about living in Fern Tree?’ and ‘What brought you to this area?’. When talking about ‘things’ participants value about life in Fern Tree, we used the term ‘features’ as opposed to ‘variables’ or ‘attributes’ as used in the previous literature (Delgado-Serrano and Ramos 2015; Partelow 2018) to describe key components of the SES that participants used to describe their neighborhood. We did this because that terminology allows for an open interpretation of both tangible and intangible ‘features’. For ease of communication, we used the term ‘community’ when referring to features within the social system and the term ‘environmental’ when referring to features in the ecological system. Features were classified, during the focus group, as belonging to the environmental theme if it is related to nature or the natural environment (e.g., mountain, wildlife, plants) or the community theme if it related to humans or human activities (e.g., tavern, bushwalking, gardening). Once a full list of features was developed, the facilitator asked participants to choose three of the ‘most important’ features, within each theme, to the community of Fern Tree and their way of life. This was either done by voting (FG1) or by agreement (FG2).

#### How are these key features related?

Individuals have a preconceived notion of how their social-ecological system operates; Moon et al. (2019) describe and demonstrate that when these notions are elicited through mental models/mapping either at the individual or group level we can glean information about how systems change. In our focus groups, participants were asked to construct a group mental model (a diagrammatic representation of how Fern Tree works) to show how the features relate in their local social-ecological system. We utilized a diagram-based activity to construct the group mental model, as it is an engaging and cognitively accessible task for groups (Moon and Adams 2016, Moon et al. 2019, van den Broek et al. 2021). We pursued a form of team mental models or ‘team situation models’ which help implicitly bring together individual mental models into one group model, thus giving a better overall sense of the dominant influence in the SES (Cooke et al. 2000; Moon and Adams 2016; Moon et al. 2019).

The group mental models were then translated into matrix form to capture the mapped links between features. The software Social Network Visualizer (SNV) was used to calculate network metrics for the each feature (nodes/vertices in a SNA) (Kalamaras 2021). SNV helps quantitatively identify the most important (focal) features of the SES, calculating both degree centrality and betweenness centrality. Degree centrality indicates how central a node (feature) is, with a higher measurement indicating more edges (links) that a given feature has with other features in the system. Betweenness centrality shows how an edge (feature) acts as a ‘middle point’ between two other features as a connecting feature or bridge; it technically measures the shortest path between two features (A to B) that must pass through a given feature. For the purposes of calculating centrality metrics, we considered only the presence of a relationship between each feature (denoted by a 1) not the nature (positive or negative). The measurements of degree centrality and betweenness centrality are calculated as below (Kalamaras 2021):

- Degree centrality measure (DC) = sum of the edges attached to a node
- Betweenness centrality measures (BC) = *the sum of δ*_*(s,t)*_*(u)/ δ*_*(s,t)*_* for all s,t ∈ V.* Here* s* is the value/location of the beginning node, *t* is the value/location of the end node, and *δ*_*(s,t)*_*(u)* is the number of all paths most pass through *u* to get from *s* to *t.*

#### What are some of the core drivers of change (in key features)?

We asked participants to talk about the way in which key features of their system had changed since they first arrived in Fern Tree, and to discuss their perceived drivers of those changes. Once a list of ‘drivers of change’ had been developed, we focused on a select few that group members thought were the most relevant and discussed their impacts on the community in both the short and long terms. Two drivers were chosen in each group: One of the drivers for each group could be considered a ‘pulse’, with immediate or very noticeable effects on a short timescale (e.g., bushfire, major developments, or tourists arriving from large cruise ships) and the other a ‘press’, with long-term effects that are not as noticeable on a day-to-day basis (e.g., population change within Fern Tree or other development pressures).

#### How might the community adapt to future change?

Participants were asked to choose two of those core drivers (one press and one pulse) and asked to show how they thought their SES map would change in response to each. When doing so, participants were then tasked with addressing on of the ‘drivers of change’ to connect it with negative and positive arrows to the rest of the SES map. They were also asked to discuss the changes, noting how they might impact their community and to consider how they might respond/adapt. The changes were considered one at a time.

#### Potential response to loss of a core feature

We also sought to understand what might happen to our participants’ SESs (their maps) if a core feature were to disappear and we focused on network metrics when doing so. This allowed us to test whether the system would be able to still maintain a similar structure and set of functions (Bodin and Tengö 2012; Kerner and Thomas 2014), in the absence of core features. We thus compare the overall size and order of centrality measures—to see whether they remain the same before and after deleting a core node (feature). ‘Core’ nodes were deemed to be those with the highest DC and BC scores. If multiple features shared the same scores, we considered qualitative information from the discussion, choosing the feature that most closely resembled an action situation.

### Populating SE-AS

Based upon our qualitative and quantitative analyses, we draw together the two focus group mental model maps of their SES systems into a single SE-AS. In doing so, we aim to piece together a mapping framework akin to that in Schlüter et al. (2019) where social (community) and ecological (natural) features create a vivid and dynamic social-ecological system.

## Results

### Features of the SES

Key environmental features identified by FG1 included the mountain, connection to nature, and native plants/wildlife; key social features included the people, outdoor lifestyle and block/land size (Table 1). For focus group 2, core environmental features included ‘space,’ wildlife, and connectedness; core community features included accessibility, community infrastructure, and the tavern). The features underlined in Table 1 are those chosen by participants as ‘the most important’ (see 10.1007/s13280-025-02338-y for full list and in-context quotes).

**Table 1:** Key features of Fern Tree (that participants like most) by focus group and type of feature (ecological, social). Underlining indicates the shortened word used to describe the ‘most important’ features during ‘mental map’ exercises

	FG1	FG2
Ecological (environmental) features	Mountain Bush Native plants and animals Bandicoots Birds (black currawongs, etc.) Climate/cold/snow Connection to nature Wilderness	Mountain (the bush) and recreation (sense of) space Wildlife Climate Connection to nature (Connectedness)
Social (community) features	Outdoor lifestyle Community (the type of people), locals and friends Block/lot size Gardening Bushwalking Recreation Pub as social hub/events Proximity to city but different feel (natural) Cultural connection Sense of place/belonging	Away from suburbia (semirural) Community, like-minded individuals Privacy and peace (homes not too close) Tavern (a hub/place) Accessibility to city life/ public transport (bus line)/other Community infrastructure Family lifestyle Playgroup (from parents and kids) Carols by candlelight (community events)

### Relationship between key features

**Fig. 2:**
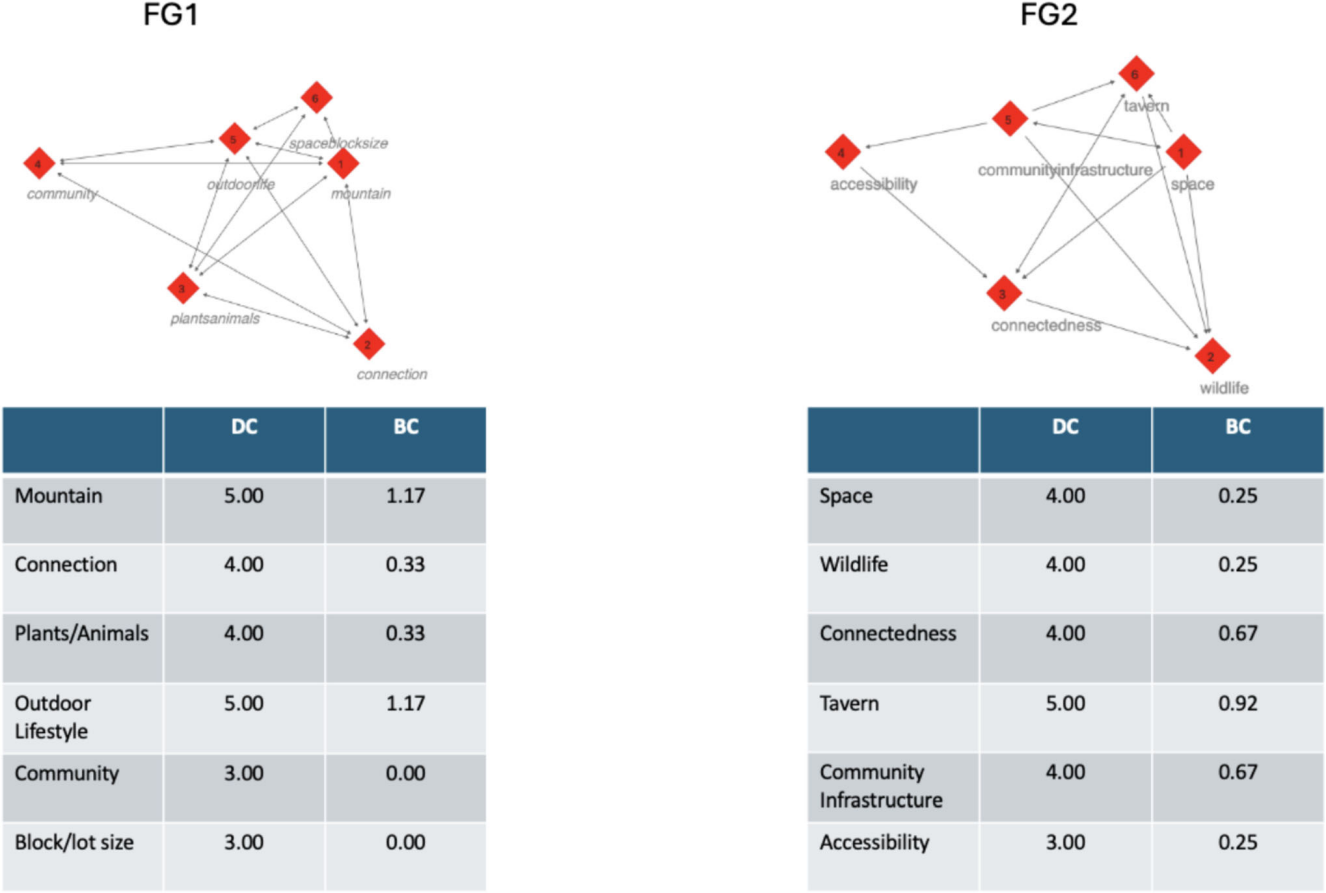
Network maps of environmental and community features for each focus group and related network metrics (DC = degree centrality; BC = betweenness centrality)

The mental model mapping activity, which focused on the six core features identified by each group, was translated into an adjacency matrix and imported into SNV to calculate the degree centrality and betweenness centrality of each feature (Fig. 2). Evidently, the mountain, as a physical anchoring feature, has the highest level of centrality as a focal feature in the network for focus group 1 with an associated community feature of outdoor lifestyle and the environmental feature of plants and animals. For focus group 2, the tavern has the highest centrality with an associated community feature of community infrastructure and environmental feature of nature connectedness.

### Core drivers of change

The core drivers of change identified by participants are listed in Table 2, which highlights the four changes that were selected for further discussion (and see 10.1007/s13280-025-02338-y for a description of all drivers, definitions, and quotes that provide context to and thus support the definitions).

**Table 2:** Core drivers of change, by focus group and type of change (pulse/pressure). Underlining indicates a chosen change

	FG1	FG2
Pulse	Losing the shop in community hub and post office Vhange in play equipment Proposed development on mountain (e.g., cable car) Changes to the pub Bushfire (actual	Change in management on the mountain (roads and cable car proposal) Better turnover in ownership of tavern Bushfire (actual)
Press	Climate change/bushfire risk Increase in tourism More families moving in (increase population/types of people) Building up homes/subdivision of property lands → clearing of bush More cats and dogs endanger wildlife Invasive weeds Inundation of evasive weeds (e.g., foxglove) Animals smaller (i.e., birds) More cars and roadkill Council not looking after place Lack of political will/lack of help from local council	Change in people (the individuals) More people (tourists) coming to the area More families moving in, leaving suburbia Lack of help from city council Local Fern Tree community leaders struggle with direction, but newsletter good Need more awareness about how to take care of the mountain and surrounding area

FG1 selected development pressure as one of their core changes (a pulse). The discussion centered around prospects of a cable car being built on the mountain. This is classified as a pulse because it could involve relatively significant change to the landscape in just a few years. The main concern expressed by participants was that the development would impact people’s day-to-day connection to nature and their notion/sense of place. Here, various topics arose from less privacy with housing development and lot sizes becoming smaller to overcrowding from tourism diminishing the sense of isolation or being ‘one with nature’ as trails and roads become overly congested. FG1 also identified a long-term concern: with the ‘type’ of people that move to the region changing over time. Specifically, the discussion focused on the fact that the lifestyles that new people bring may be quite different to those currently living here which may affect their well-being especially if the new residents are not as attuned to Fern Tree’s connection to nature as current residents are. Here a main concern expressed by participants was a change in demographics from those moving into the area from city life and not understanding what living in the area and as a part of nature entails.^1^ It is classified as a press, because it could take up to a generation for attitudinal and lifestyle changes such as this to impact current residents.

FG2 selected changes to tavern ownership as a core change (pulse). It is classified as a pulse because a change in the ownership of a business can happen quite quickly and therefore the overall operation of such a place. As of now, the tavern provides a place for community, for both locals and tourist alike to come together in a very hospitable environment to enjoy the community and nature that Fern Tree provides. FG2’s selected press, concerned increases in tourism/visitation from outside the region, is something that has been steadily increasing over time. Here, the discussion focused on the fact that a gradual increase in the number of tourists coming may slowly degrade the lifestyle and environment of those living in Fern Tree. In addition, housing developments were a concern, with participants noting that they may not appeal to the kind of people that care as deeply for the land as those currently living there but being cautious to recognize that it is the next generation who will ultimately make a place.

### Adaptation to change

**Fig. 3:**
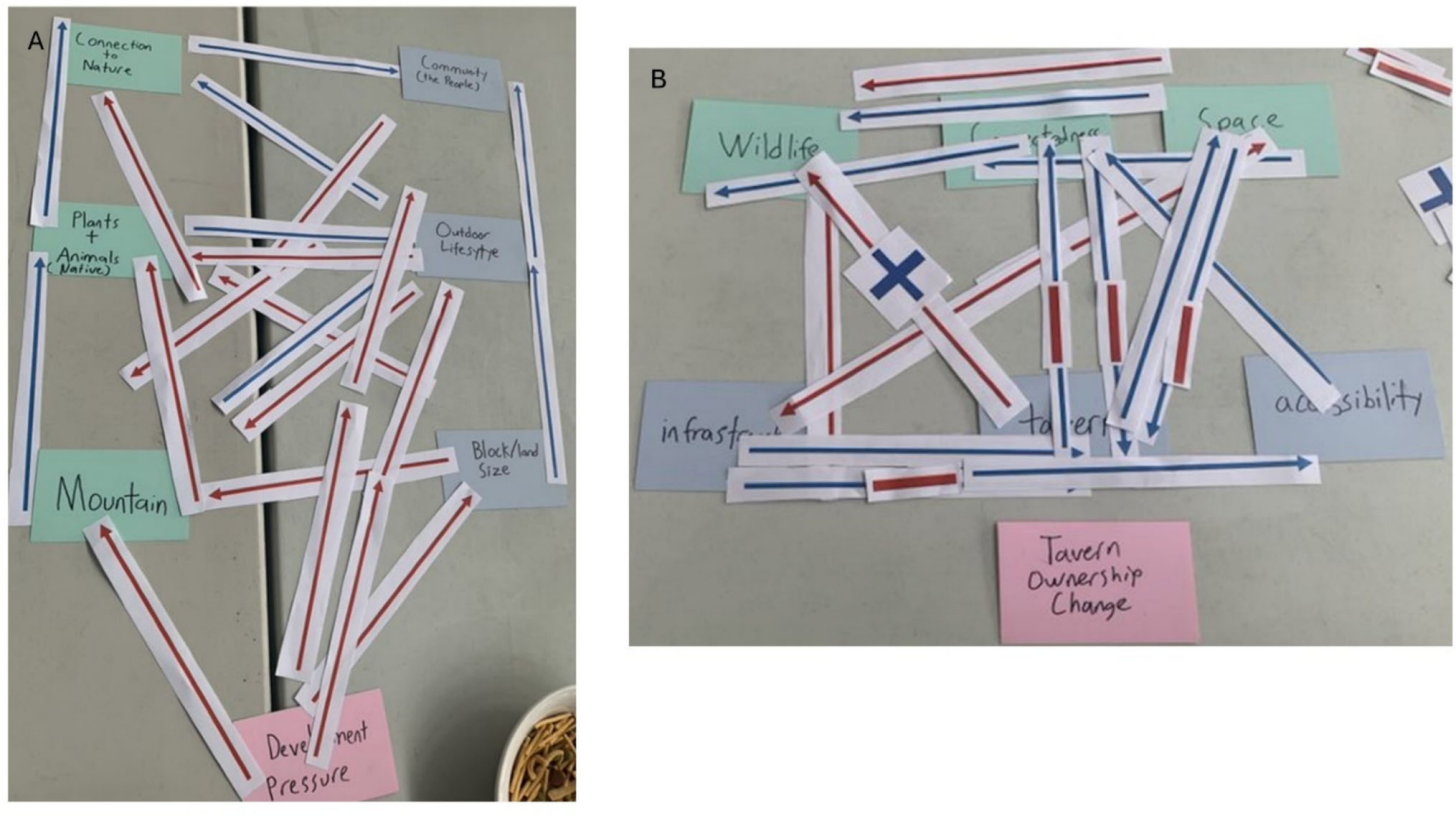
Mental maps for **A** focus group 1 as they discussed how they might adapt to development pressures and **B** focus group 2 as they discussed how they might adapt to change of tavern ownership

The mental maps used in both focus groups clearly show that participants felt core changes would impact all key features of the SES, either directly or indirectly, in positive or negative ways. See Fig. 3 as an example of how they mapped changes in the networks as they respond to drivers of change: ‘development pressure’ and ‘changing of tavern ownership,’ respectively.

The thematically coded discussions relating to adaptation highlight that participants of both focus groups were forward-looking and had generally positive outlooks for the future (10.1007/s13280-025-02338-y). They were able to navigate and respond to the prospective changes that may arise and find an adaptive outcome (i.e., resilience). This capacity demonstrates their ability to use local resources (features) to find ways of building resilience and maintaining well-being. For example, when looking at a potential pulse, such as development of the mountain via a cable car, some FG1 participants were able to make light of a new perspective and thought of new ways to make change of what first appears to be a difficult situation.

‘I'd be a little bit open minded and think maybe they'll close the road as a result, and I would really like that…[putting in] electric buses up the road…’—FG1 participant.

When looking at a potential press, such as the changing type of people and demographic shift, some FG1 participants were able to make light of a new perspective and look toward the future what first appears to be a difficult situation.

‘ This next generation is coming for it. So yeah, that's right. It's for them to address’ —FG1 participant.

A potential pulse that can drive change is that of tavern ownership and how that affects the overall milieu of the community as one participant from FG2 describes what might happen if ownership goes from good to bad: ‘So if the owners change and they wanted to do things completely differently, although bad business people, and so forth, could really change the connectedness of the community, people getting together could change the infrastructure because we wouldn’t have any way to go eat and have coffee or just to sit outside and meet people and so forth.’

### Loss of a core feature

**Fig. 4:**
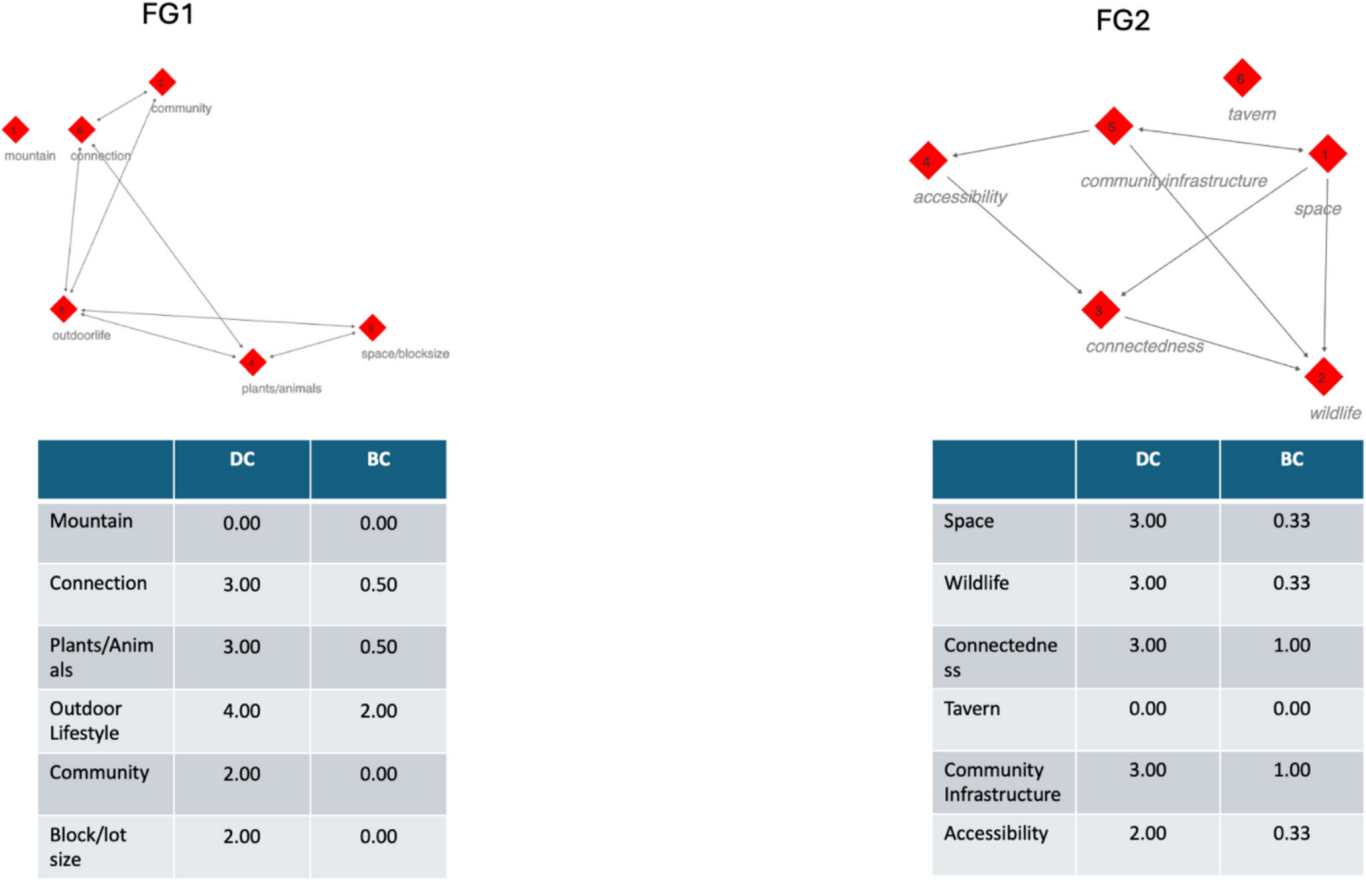
Focus group results of direct centrality and betweenness centrality tests with key feature removed

Figure 4 displays the network generated in SVN along with the degree centrality and betweenness centrality calculations without the mountain (FG1) and the tavern (FG2). For FG1, we see that outdoor lifestyle retains numerous connections and remains highly central in the overall network (DC = 4 and BC = 2). Moreover, outdoor lifestyle increases its BC score from 1.17 to 2, higher than the tavern’s BC of 1.17 before removal, thus indicting a sign of a resilient system in that the system retains structural connectivity, and outdoor lifestyle maintains connections with other nodes even in the absence of mountain. For FG2, we observe that with the tavern gone, connectedness and community infrastructure become the most central features with (DC = 3 for both) with betweenness values increasing from 0.67 to 1 each. As with focus group 1 results, we see that the network is resilient to the loss of the central feature (in this case tavern) as all other features remain similarly connected (DC metrics) and with highly central adjacent features maintaining betweenness connections.

### Social-ecological action situation

**Fig. 5:**
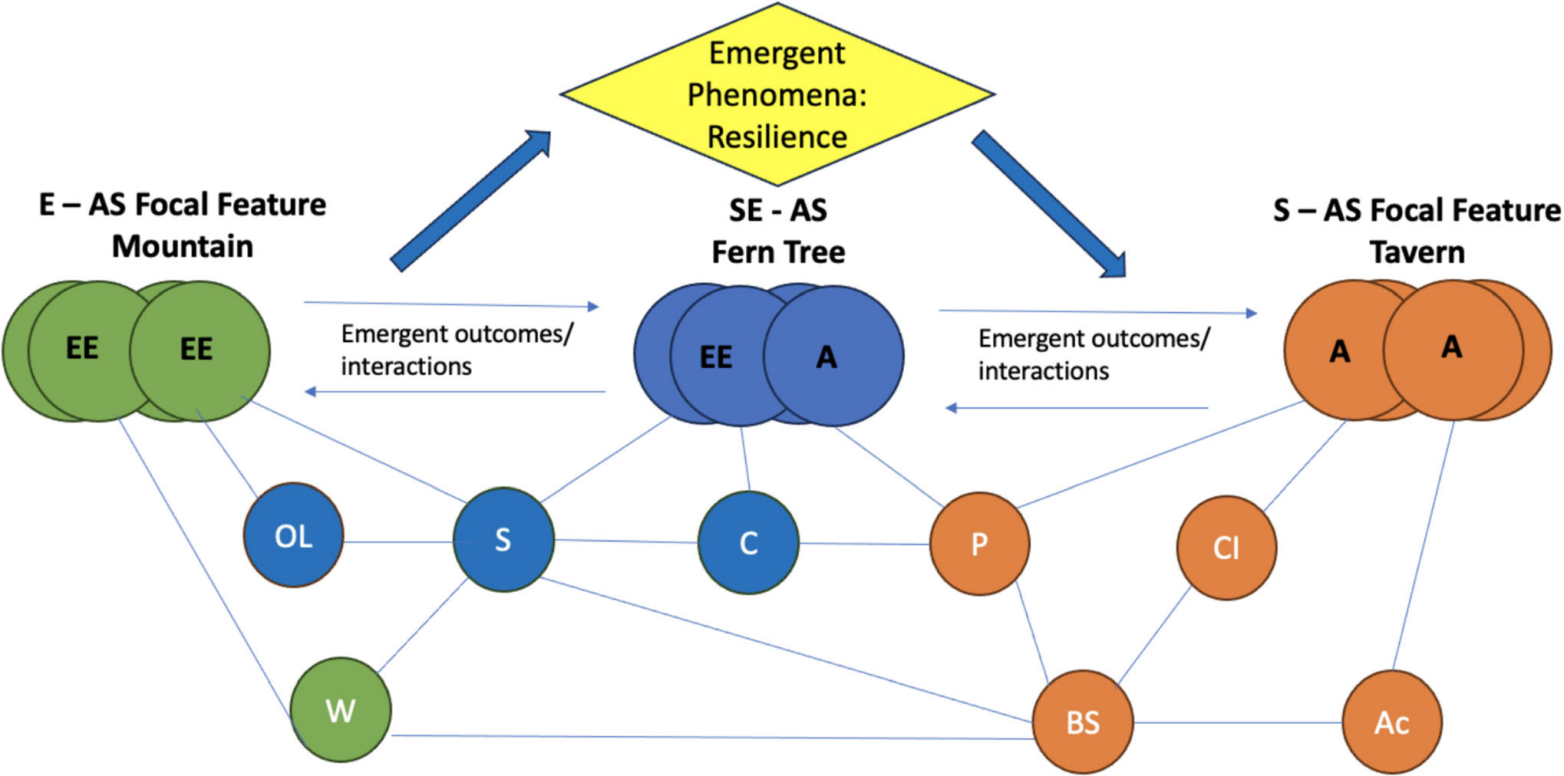
Fern Tree SES involving the emergent of a SE-AS. An illustration of how focal features form the main action situations (ecological and social) help form resilience in Fern Tree with the backdrop and influence of supplemental features that support (and, if necessary, help replace) focal features and action situations, particularly in the face of presses or pulses that might erode existing AS. Green—a predominant environmental influence, orange—a predominant community influence, blue—hybrid/ a strong combination of both environmental and community influence. Components in the figures are: EE—ecological elements, A—actors, W—wildlife, OL—outdoor lifestyle, S—space, C—connectedness, P—people (locals), BS—block size, CI—community infrastructure, Ac—accessibility

Figure 5 displays combined focus group SES mental maps in which focus group one focused on the ecological action situation (E-AS; anchored around the mountain) and focus group two focused on the social action situation (S-AS; anchored around the tavern). Where factors of the SES were shared across the two focus groups (e.g., connectedness, space), these have been placed within the SE-AS figure based on the strongest relationship (quantitative social network metrics and qualitative thematic evidence).

## Discussion

In our study, we sought to pilot methods to elicit local community mental models of their SES and to combine these with network analysis to learn more about what makes local communities ‘resilient’. The pilot included just a small number of participants, so empirical results cannot be generalized across the community, but the methods are clearly able to generate new and useful insights, particularly about social capital, redundancies, and adaptive capacities—all critical parts of resilient systems.

The focus group discussions and team activities encouraged participants to identify and map relationships between core social and ecological features in their neighborhood and to identify and discuss drivers of change into the future. Though there were many drivers listed by each focus group for both presses (long term) and pulses (short term), achieving collective agreement on certain features is indicative of the start of a bottom-up approach that addresses concern in a community that can later be part of a more cohesive polycentric governance that works across scales addressing societal problems (Ostrom 2005). More importantly, this method sheds light on place-based research by aiding our understanding of the dynamics between local and global sustainability goals and SES research (Balvanera et al. 2017; Berthet et al. 2022).

Our focus group activities, together with the associated network analysis, highlight the importance of the key features to the community. For this pilot, these included the mountain (FG1) and the tavern (FG2). Other features discussed within the focus groups (wildlife, connection to nature, block size, accessibility, public infrastructure, outdoor lifestyle, and space) appear to be supplemental to the core. Supplemental does not, however, mean unimportant. Our approach also allowed us to see what would happen when a focal feature was removed, finding evidence (both quantitative and qualitative) to suggest that both focus group systems have redundancies (specifically, other features) that stepped in to replace missing nodes. Such redundancies in features are at the heart of what it means to be resilient as discussed by Walker et al. (2004)—suggesting that our method is able to generate information that helps assess the resilience of systems.

In our pilot test, measures of connectivity (specifically, of betweenness centrality) increased for remaining features when the most significant core feature was removed; outdoor lifestyle replaced the mountain in FG1, and connectedness and community infrastructure replaced the tavern in FG2. Systems that are able to rebuild/create new strong connections when parts of the system are removed are generally resilient. The network analysis and related focus group discussions thus suggest that the supplemental features may provide a means to maintain sustainability and resilience—particularly given the participants’ deep knowledge of the system and their willingness to learn together. Evidently, loss of core features does not necessarily lead to loss of overall well-being since supplemental features can replace them, allowing participants to continue to enjoy the things they love most about living in their community (see supplementary materials and Table S3 for further analysis).

Our results suggest the presence of an overarching social-ecological action situation (SE-AS), as described in (Schlüter et al. 2019), with ‘community resilience’ being an emergent outcome. Each group focused on a different aspect of the SE-AS: one on the ecological side (FG1) and the other on the social side (FG2); together, they suggest a robust system and provide insights into how resilience can be maintained and built at the community level in Fern Tree (Fig. 5). The two focal features create action situations with the ability to build long-term sustainability and resilience. This is done in two ways, one, by ecological elements (EE) or the benefits that the mountain offers (living in nature, weather, etc.) which creates the ecological—action situation (E-AS). Second, the actors (A) that interact within the Tavern domain and derive benefits from that which creates the social action situations (S-AS). These actions situations interact among themselves and merge with each other, thus creating a social-ecological action situation (SE-AS). It is through these interactions that an emergent phenomenon (outcome) occurs from the creation of the SE-AS, this being potential resilience in Fern Tree, similar to what Herzog et al. (2022) discover with the emergence of improved lake integrity despite challenges differentiating action situations across the governance system and its respective scales.

The above observations generate a novel and evolved concept adapted from Schlüter et al. (2019) which allows the other ‘ecological and actor entities’ shown in Fig. 5 to be more than just inputs (or participant) in the SE-AS. What determines whether a system, and more specifically a SE-AS, is resilient, is its ability to keep the system (locale) stable, and thus sustainable, in the face of change(s). The supplemental features described by the participants and shown in the lower part of Fig. 5 have a key role to play since they are, in essence, forms of redundancies, as described in Norris et al. (2008). Future research could usefully test whether these approaches are also able to identify redundancies and emergent outcomes in different contexts.

In sum, our results thus suggest that if our focus group participants were to experience a shock (or pulse change) that removed one of their core features, a new focal AS could emerge, created from the supplemental features. This helps to maintain the overall social-ecological action situation and desired outcome of resilience, i.e., consistent lifestyles in Fern Tree, demonstrating how the SES can be seen as a complex adaptive system, where the interacting parts are more important than the whole. In the subsections below Fig. 5, we leverage insights from the study to further discuss how those core characteristics underpin the system’s operation.

### Redundancies

Redundancies are an integral part of a resilient system—with different features providing similar functions and outcomes, so that one can come in and replace that of a waning feature (Anderies et al. 2004; Walker et al. 2006). Focusing first upon the mountain, the conversation in FG1 focused on nature and the connection that people have to the land and place—both physically and spiritually. The mountain was the focal/core feature—the ecological action situation (E-AS). It stood out as a beacon of hope; an overarching presence, not just in Fern Tree but throughout all of Hobart—an understanding that the mountain will still be there for future generations to benefit from. Though there was some trepidation about short-term development and long-term demographic change, the participants were able to visualize other futures or pathways backed up by other supporting environmental and community features of value (i.e., outdoor activities or native wildlife/plants) that help them stay grounded and resilient in place. Discussions also highlighted dynamic changes, and adaptations, over time that may help explain underpinning processes that support resilience—similar to changes observed by Roux et al. (2022) in their study of forest inhabitants of Europe and Asia. It seems that a symbolic, coevolution process has occurred between humans and nature (i.e., Fern Tree residents and Kunanyi/Mt. Wellington)—with workshop participants suggesting that their spiritual connection to the mountain has grown stronger and deeper over time. Though the mountain cannot be physically removed, it evidently provides a deep connection to other features of value such as wildlife and bushwalking. If some of what the mountain provides to humans (e.g., opportunities to walk in one area) is eroded by human intervention, then participants discussed how they would find new ways to connect to nature and therefore the physical mountain. Here, the mountain likely plays the role of an ecological action situation—providing a means to resilience and sustainability building through natural features.

The tavern was mentioned in both focus groups but played a much larger role as a chosen community feature in FG2. The tavern plays a significant role as a community hub, bringing both locals and tourists together. This reinforces the role that the tavern may play; similar to what Cabras and Mount (2017) find in rural Irish pubs that act as a ‘third place’ a bridge between one’s home (first place) and workplace (second place). Accessibility acts as a complement, and with its proximity to Hobart, the tavern can act as a bridge that brings people in contact with not only Fern Tree but also nature. Thus, the tavern likely plays the role as a social action situation: another building block for resilience and sustainability and the second part of a broader social-ecological action situation.

### Social capital and adaptive capacity

Social capital and adaptive capacity are also critically important for resilience (Dressel et al. 2020). It is through a community’s adaptive capacity and ability to create social capital (Auckland and Kilpatrick 2021) that communities are able to maintain well-being and connection to place in the face of short-term threats (pulses) and/or long-term challenges (presses). See, for example, Dressel et al. (2020) who highlight the importance of adaptive capacity across multiple scales for governance; Ross et al.(2010), Adger (2000) and Adger et al.(2009) who make clear the importance of adaptive capacity and social capital for resilience; Fath et al. (2015) who highlight the importance of social capital when navigating the adaptive cycle of change; and Harangody (2022) who show its importance for communities affected by flooding on the island of Kaua’i.

Social capital and adaptive capacity are not only relevant by and for themselves but they allow communities to develop redundancies in their SES (Norris et al. 2008), thus ensuring that supplemental features can replace ‘core’ features thus maintaining function and therefore resilience(Adger 2000; Collins et al. 2010; Folke 2016). It is through dynamic processes that people have the opportunity to learn about and develop new ways of living to maintain well-being (Preiser et al. 2018). Ross et al. (2010) and Berkes and Ross (2016) suggest that community resilience is strongest when residents are able to recognize the importance of the interconnectedness of the ecological and community realms and their respective features. Gallopin (2006) further suggests that communities are able to build resilience despite facing possible vulnerabilities that come about by building strong adaptive capacities.

The focus group participants were very comfortable talking about key features, connections, changes, and adaptations. The methods used to build the mental maps were similar in nature to the methods used by Johnson et al. (2022) when constructing SES maps for Alaskan national parks, who report that their participants, were able to create fuzzy cognitive maps that show an innate understanding of the interrelationships of ecological, social-cultural, and social-economic features that thus create an understanding of a local SES. Our focus groups demonstrated a similar deep understanding of complexity and innate working knowledge of their SES when building a shared mental model; their associated discussions showed willingness to learn from others. These two observed characteristics (knowledge and willingness to learn) together demonstrate both resilience and a capacity to further strengthen resilience (Amundsen 2012; Patel et al. 2020; Auckland and Kilpatrick 2021). These are critically relevant characteristics since it is the response of people and community to change that builds, or detracts, from resilience.

In the face of potential future adversity, focus group participants were able to foresee a pathway forward as they responded to potential shocks and presses. Redundancies within the system, related to core SE-AS, allow for this to happen. The tavern facilitates knowledge sharing, possibly around numerous potential changes—e.g., when thinking about bushfire risks, potential impacts, and strategies (Lucas et al. 2022). If the tavern were to deteriorate in quality so that residents no longer used it as a place for knowledge sharing, then redundancies in the system could be used instead, e.g., the community center (community infrastructure) could step in and fill the role. Similarly with the mountain, if a cable car were built or more visitors deteriorated the quality of current mountain experiences, then there would be other outdoor lifestyle activities that could be substituted. This suggests that the participants were at least in some sense, ‘resilient,’ being aware of their surroundings and being able to navigate different phases of change (the adaptive cycle) where there are various aspects of change waxing and waning through various stages across time (Fath et al. 2015).

## Conclusion

Our research innovatively combined focus group discussions and team mental modeling with network analysis to study a SES in the peri-urban neighborhood of Fern Tree, near Hobart in Tasmania, Australia. This pilot demonstrates the utility of focus group methods to rapidly elicit mental models as the basis of network analysis to evaluate the potential redundancy and resilience in SES. Our analysis suggests the presence of an overarching social-ecological action situation (SE-AS) (Schlüter et al. 2019) with ‘community resilience’ being an emergent outcome. The ‘evidence’ for resilience relates to the presence of redundancies in core features and the group discussions which demonstrate the presence of social capital and adaptive capacity within the community. Our analysis suggests that if focus group participants were to experience a pulse that removed a core feature, a new focal AS could emerge, created from supplemental features, thus maintaining the overall social-ecological action situation and consistent lifestyles. Findings were consistent across focus groups and for both the qualitative and quantitative analysis of results. Building sustainability and resilience requires playing the long game and the ability to navigate cycles of change. Fern Tree seems to be on that path and gleaning from the focus group participants as the adage states, ‘the more things change, the more they stay the same’.

The small scale and scope of our study are such that our empirical findings cannot and should not be generalized. This work is best considered a ‘pilot study’—providing a prototype or ‘proof-of-concept’ demonstration of a novel way to use network analysis to improve our understanding of factors that contribute to resilience in small-scale SES. Further similar research could improve our understanding, but this study demonstrates the value of using a social-ecological systems (SES) approach to learn about a community. It enabled us to conceptualize how factors within an SES relate and change over time, providing a solid foundation for growth and development that builds and maintains resilience. We extended available SES frameworks, drawing together key aspects of different frameworks and then mapping core SES components (features), to learn how they relate, and whether the system is resilient to presses and pulses. This methodological approach laid a foundation and helped shed light on the social adaptations that occur among the dynamic interactions that happen in SES—and could potentially be used in numerous other contexts to further enhance understandings of resilience at nested scales from local, to state and national levels.

## Supplementary Information

Below is the link to the electronic supplementary material.

Supplementary file1 (PDF 674 KB)

## Data Availability

Data presented within this study was collected under human ethics approval (ethics approval H27215). Under ethics, all original data can only be accessed by the primary researchers and cannot be shared. All summarized data relevant to analyses are presented within the main text and supplementary materials.

## Footnotes

1 For demographic reference, as of 2021, 50% of Fern Tree residents (763 total) are over the age of 45, 305 private dwellings with 227 families (avg. 1.9 children), a median yearly income of 103 480 AUD. Compared to 2011, 44% of residents (666 total) are over 50, 286 private dwellings with 204 families (avg. 1.7 children), a median yearly income of 77 688 AUD. https://abs.gov.au/census/find-census-data/quickstats/2021/SAL60199.
