# Supplementary material for: Network analysis can provide useful insights for building resilience in social-ecological systems
Source: Ambio. 2026 Feb 27;55(8):1882–97. doi: 10.1007/s13280-025-02338-y (PMC13319644; doi:10.1007/s13280-025-02338-y)
Supplement: Supplementary file 1 — Supplementary file1 (PDF 674 KB) [file 13280_2025_2338_MOESM1_ESM.pdf]

AMBIO: Supplementary Information

Article title: Network Analysis can provide useful insights for building resilience in social-ecological systems

Authors: Paul Doehring[1*], Vanessa M. Adams[2], Natalie Stoeckl[1,3]

1 Tasmanian School of Business and Economics, University of Tasmania, Hobart, Australia

2 School of Geography, Planning, and Spatial Sciences, University of Tasmania, Hobart

Australia

3 Centre for Marine Socioecology, Hobart, TAS, Australia

*** Corresponding Author email:** paul.doehring@utas.edu.au

Supplementary Material

Table S1- Definitions of Key Features and quotes that help contextualize. The focus group is provided in () for each feature

Key Feature Definitions	Quotes
**Mountain (FG1)**<br>- Forested area providing space for nature and recreation<br>- overarching presence across community boundaries in Fern Tree and greater Hobart<br>- unique and symbolic presence with respect to indigenous knowledge	"I think like when I get up in the morning and I see the mountain they just sort of feel happy and I just it's worth sort of feel like it's a guardian sort of looks after my mental health"-<br>FG1 participant
**Space (FG2)**<br>- a sense of place<br>- one can get lost in nature, but also still be in a city<br>- an overall feeling with neighbours of camaraderie around nature	"The mountain was the big drawcard probably for us in terms of recreation and other opportunities on the mountain and just having that sense of space and yet not being on top with your neighbours…that sort of escapism, making consensus of space and obviously then that connects to the nature, wildlife, plants, everything that comes with that" – FG2 participant
**native plants and animals (FG1); Wildlife (FG2)**<br>- observing native plants and wildlife (wallabies, pademelons, bandicoots, birds, etc.)	"I see how the wildlife reacts to the seasons. And so I know what season is according to what the birds are doing for example" – FG2 participant
**Connection to Nature (FG1);**<br>- aware of one's surroundings (both spiritual and physical)<br>- the interactions one has with the environment on a day-to-day basis	"…it's sort of what happens to the place happens to you. That sort of sense. embedded in it. That it's kind of it's part of you and you're part of it. And you know, I could live other places but I would lose that that connection to place"-<br>FG1 participant
**Connectedness (FG2)**<br>- people of similar thinking; living and understanding the connection to nature<br>- belonging to a community	"…belonging to the community and you know, I think where you live we really affects that sort of connectedness depend on what's going on around you and the stresses out not stresses personal says business stresses out in the community." – FG2 participant
**Outdoor Lifestyle (FG1)**<br>- what one can do in Fern Tree (gardening, bushwalking, mountain biking, birding, etc.)	"I love outdoor activities and this is the place to do it …The mountain is on our doorstep…You can go bushwalking we're into bushwalking and bike riding. Both mountain biking [and] road biking, and…the other box it ticks for us is the garden we're really into our gardening" – FG1 participant
**Community (the people) (FG1) -**<br>- those that live in Fern Tree, the interactions that come from that with neighbours and community events	"Fern Tree tick[ed] the box for me because of the space and the sense of community as well" -<br>FG1 participant
**Block/land size (FG1)**<br>- the land on which one's home sits on<br>- the space between neighbours and the privacy that comes from that	"I want my kids to have space. I want them to have land. And so Fern Tree ticked all the boxes for us" –<br>FG1 participant

**The Tavern (FG2)**<br>- a main source of community – a hub<br>- a place for both locals and tourists	"I think the recent current owners of the tavern has also made it a focal point for the community. And because they've done really well to the tavern, they've actually made it a really comfortable place to go to and there's lots of tourists who's come by… and go for coffee or whatever. Bowl of chips or something that I think that's really important." – FG2 participant
**Accessibility (FG2)**<br>- quick access to Hobart CBD and other nature reserves (i.e., Waterworks)<br>**-** able to still enjoy city life	…"you've got that accessibility I suppose that accessibility makes you feel connected to things outside of Fern Tree" [the city (Hobart)] – FG2 participant
**Community Infrastructure (FG2)**<br>- community hubs (community centre, playground, BBQ shelters, etc.)<br>- housing development/block size	"And I was so excited when because I will probably at that age when I went to playgrounds where it was going to be done up. I don't know who they interviewed to do it, but they should have interviewed children to be about two years in the making. It's not suitable for older children or younger children" – FG2 participant

Table S2- Drivers of Change by focus group with definition (as provided by participants) and contextual quotes

Focus Group	Term used to describe, and type of change	Definition (from participants)	Contextual quotes
**Focus Group 1**	Development Pressure - (Pulse)	Increase in infrastructure and density for commerce e.g. tourism (cable car)	"I think also on the mountain, there's been a fair bit of pressure in terms of recreation is sort of mountain biking this there's been quite a bit of development of, of mountain biking tracks, and then over people doing some digging and doing trying to do a good job to minimize erosion and stuff like that." – FG1 participant
	Changing Type of People - (Press)	General change in demographics, people who may not care as much about the Community	"…I think more people would have sort of changed over the years, perhaps more families … moving up here which is obviously what we've been discussing about community lifestyle." – FG1 participant
**Focus Group 2**	Change in Tavern Ownership - (Pulse)	When the tavern which currently has a great ownership changes to something like in the past (poor ownership)	"…they really have turned it around. After a lot of years, so failed. ownerships and not much of a community feel." – FG2 participant
	Increase in Demand (Visitation) – (Press)	The overall pressure of tourism and new housing/resident moving into the area	"…I've got a feeling the people who are fleeing suburbia coming up here know that that's one of the last 12, 30-40 years…" – FG2 participant

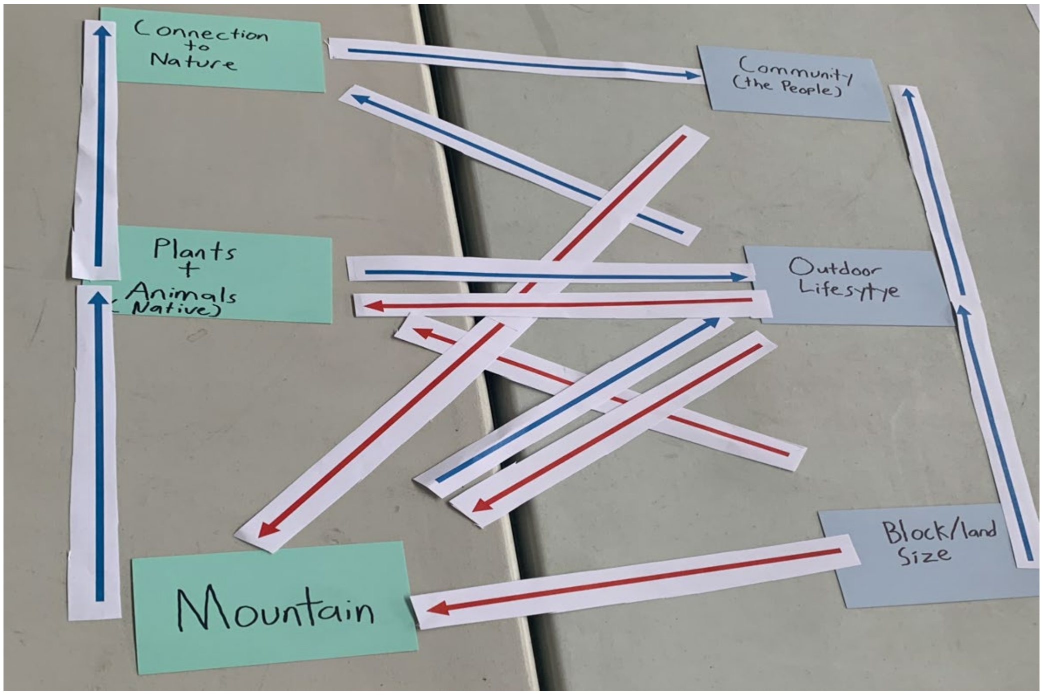

Fig. S1 *Mental mapping of FG1 local SES*
Green notecards represent the environmental features and blue notecards represent social features. The blue arrows represent a positive relationship from one feature to another and the red arrows represent a negative relationship between one feature to another.

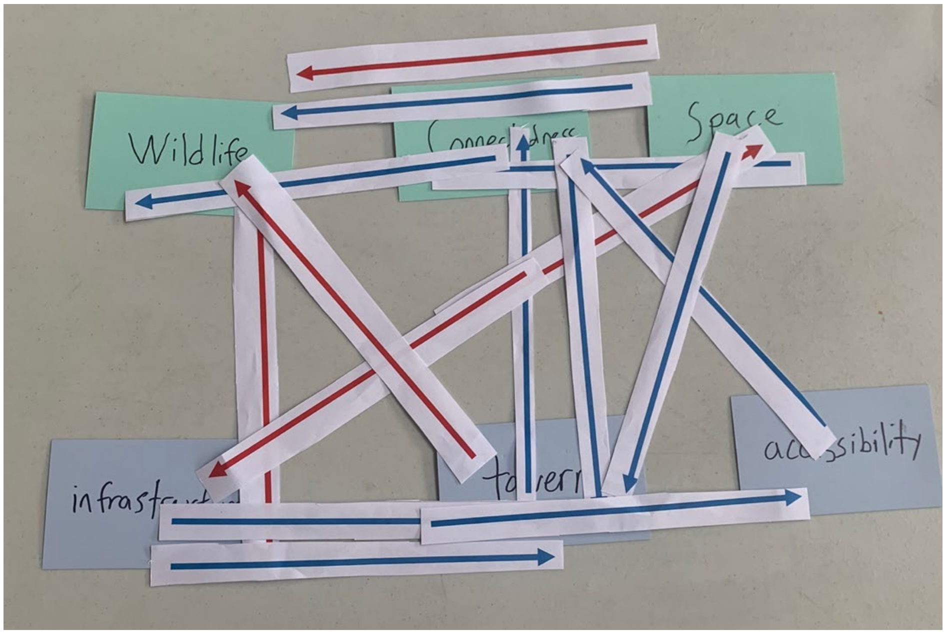

Fig. S2 – *Mental mapping of FG2 local SES.*
Green notecards represent the environmental features and blue notecards represent social features. The blue arrows represent a positive relationship from one feature to another and the red arrows represent a negative relationship between one feature to another.

FG2 did not connect the driver of change notecards to the rest of the SES and in liue of rearranging the arrows from the initial SES map, they used pluses and minus signs to demonstrate how the system diminishes or amplifies. Supplementary Figures 5 and 6 display the map change after implementing the drivers of change: ‘tavern ownership’ and ‘increase in demand(visitation)’ respectively.

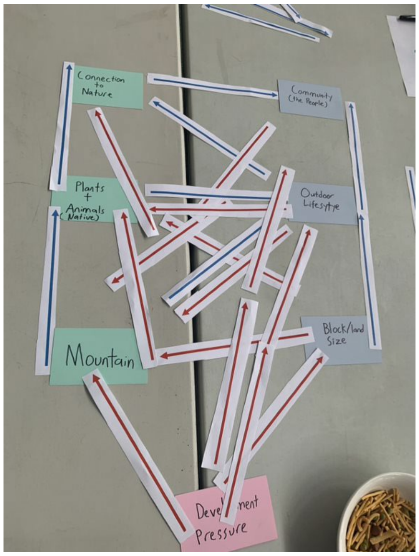

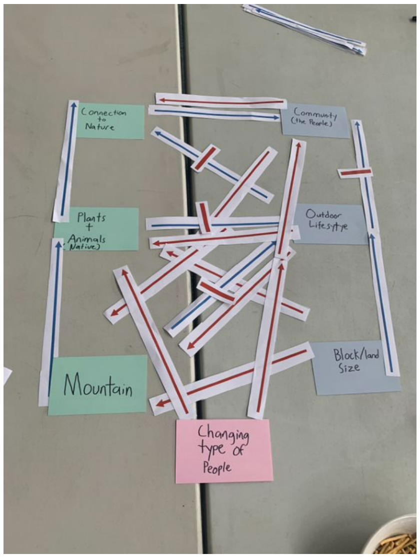

Fig. S3 - *FG1 first driver of change (pulse - development pressure)*

Fig. S4 - *FG1 second driver of change (press - changing type of people)*

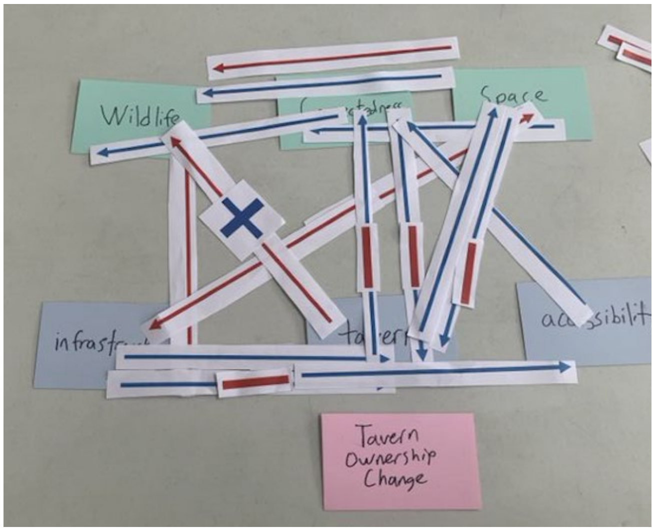

Fig. S5 - *FG2 first driver of change (pulse - change of tavern ownership)*

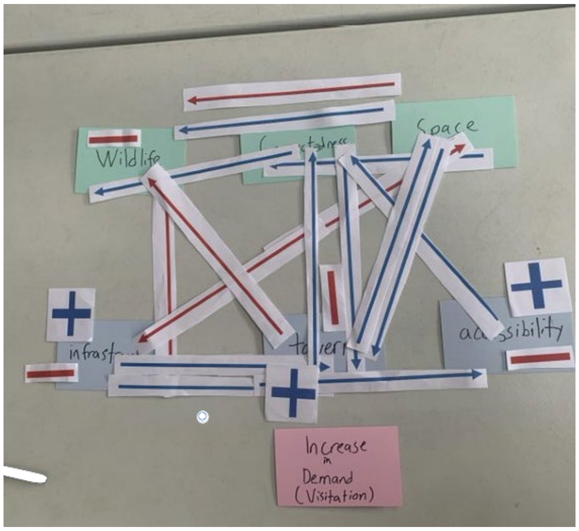

Fig. S6 - *FG2 second driver of change (press - increase in demand/visitation)*

Table S3 – Qualitative responses to Change (Presses and pulses).

Responses are grouped by focus group and aligned to the four questions asked of them. Italics represent general takeaway themes and indicative quotes are provided for each theme.

**Questions**	**What would be your initial reaction to this change? How would you respond/react?**	**How do you think others in Fern Tree would react/respond initially?**	**Do you think Fern Tree will get back to exactly the same state or be slightly different?**	**How would you and the community evolve over time?**
**FG1**				
**Driver #1: Development Pressure**	*Disappointment, but adaptive, solution/response driven*<br><br>"I'd feel sick."<br><br>"I'd be a little bit open minded and think maybe they'll close the road as a result, and I would really like that… electric buses up the road…"<br><br>"If there was something like that to happen here. That didn't directly impact on the community. So I'd be more inclined to be more active and more vocal and	*Future focus, capabilities of next generation*<br><br>"So obviously be open minded that it's actually the next generation's decision"	*Re-development of past historical sites*<br><br>"we're looking at it would be good to see something developed with the springs where we could bring community people"	*Mixed responses: adapt with change or leave*<br><br>"And it would it would make me feel kind of…sad, you know, looking at it. Would it make you leave?...Possibly. Probably not, but it would could be something that might contribute to a decision to leave."<br><br>"It might be the third strike with climate change, saying it's time to move and it would break my heart."

Questions	What would be your initial reaction to this change? How would you respond/react?	How do you think others in Fern Tree would react/respond initially?	Do you think Fern Tree will get back to exactly the same state or be slightly different?	How would you and the community evolve over time?
	more organized and more aggressive standing up for this place.”			
**Driver #2: Changing Type of People**	*Become more active, find solutions*<br><br>“But I think you can start to counteract changing culture by just being more actively involved in yourself…trying to contribute to the community that you want it to be rather than being unhappy about it changing …”<br><br>“…you can like through the community newsletter. You can let people know what's happening. Invite them in. “	*Deal with the change*<br><br>“As we know, people do make or break a place…”	*Look forward to change*<br><br>“…could be a positive it could be lots of people who care about well, we suddenly realized what a great place Fern Tree to live because of the place yeah we don't want to change it. We want to be here just to enjoy it. And be custodian of it. And that would be fantastic.”	*Learn to live with change and adapt*<br><br>“This next generation is coming for it. So yeah, that's right. It's for them to address”
**FG2**				

Questions	**<u>What would be your initial reaction to this change? How would you respond/react?</u>**	**<u>How do you think others in Fern Tree would react/respond initially?</u>**	**<u>Do you think Fern Tree will get back to exactly the same state or be slightly different?</u>**	**<u>How would you and the community evolve over time?</u>**
**<u>Driver #1:</u> Change in Tavern Ownership**	*Loss of community*<br><br>“So if the owners change and they wanted to do things completely differently, although bad business people, and so forth, could really change the connectedness of the community, people getting together could change the infrastructure because we wouldn't have any way to go eat and have coffee or just to sit outside and meet people and so forth. “	*Loss of community*<br><br>“It would change the space things because you know that that is the space that you go to. It's sort of like a community space where we're where people get together and sit and sit and sit in the sun is sort of it's a nice community space.”	*What is lost:*<br><br>“I think what the tavern does or potentially does is it does bring people together potentially. And they because there's the bigger chance of people getting together and meeting people who live up here and then developing empathy with those other people and understanding why some people get crazy about weeds or for example”	*Go with the flow, aim for the best, something else will take its place*<br><br>“I think it is, I think the tavern is symbolic of what when you get the right people involved, something that can work really well. And just make you know, for good health for good people come to attract tourists who, who drive down the mountains and see the sign for the tavern and cup up have coffee or slice a cake or so forth. I think it's really symbolic for something that has done well and can serve the needs of the community can serve the needs of tourists, but it isn't big and ostentatious.”

Questions	What would be your initial reaction to this change? How would you respond/react?	How do you think others in Fern Tree would react/respond initially?	Do you think Fern Tree will get back to exactly the same state or be slightly different?	How would you and the community evolve over time?
**Driver #2:** **Increase in Demand (Visitation)**	*Disappointment/off-putting*<br><br>"Think a lot of people put me off because that's why you choose to live. Live here that we're not as densely populated. Yes, because it can take away from that experience. Or it's going to put pressure if you're trying to choose other areas to go to for solitude. Or your recreation time."<br><br>"…you could get the wrong type of people wanting to come live in Fern Tree thinking it's an idealistic world, when it's not always an idealistic world and you have to be involved in	*Mixed bag: can spur care or negligence*<br><br>"the mountain bike tracks are positive and negative that are positive because they take more people over the mountain. And they, you know, once again, they get people more appreciating the amount of what it is and but it could be seen as a negative because we're having to cut new tracks, new people, the density of the people not knowing that not everyone's going to use the mountain are going to leave their stuff behind in their lives. So there are positive and negative negatives with the increased demand or visitation on the mountain."	*Education and more council involvement needed to bring about quicker recovery*<br><br>"I suppose it's then its education, understanding things…"<br><br>"I think almost needs to be council lead, as in, you know, that council just needs to get, you know, talk to people up here and discuss the awareness kind of what's going on in the yards and environments around them and how that affects the community as a whole."	*-Go with the flow*<br>*-External risks to development*<br><br>"But I wonder if 10 or 20 years time, whether they'll that will change again and people will want to live differently in 10 to 20 years time and maybe they'll go back to wanting to live in suburbia or something like that. And I wonder if that will happen over time and I I also think that there's a natural ceiling on the amount of people that can live in Fern Tree because of where we're boxed in."<br><br>"I'm very concerned about the amount of fuel that's on there as a resident and that's probably the reason why you would move not because we don't love the mountain but because we're scared that we'll lose with one fire which is going to happen it's not saying that lose everything that you've got."

Questions	**What would be your initial reaction to this change? How would you respond/react?**	**How do you think others in Fern Tree would react/respond initially?**	**Do you think Fern Tree will get back to exactly the same state or be slightly different?**	**How would you and the community evolve over time?**
	the community or you had to be involved in environment around it for example”			
